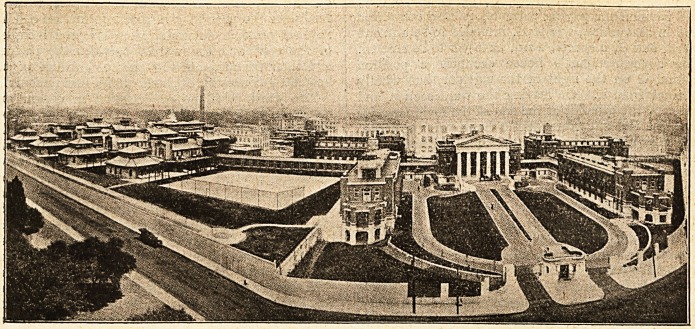# The Peter Bent Brigham Hospital, Boston

**Published:** 1916-12-30

**Authors:** 


### THE PETER BENT BRIGHAM HOSPITAL, BOSTON.

The founder of this hospital, Peter Bent Brigham, died in Boston, Mass., May 24, 1877, and this date has been reserved ever since as Founder's Day by the Corporation formed on May 8, 1902, to carry out the founder's will by providing that the residue of his property should be left to accumulate for twenty-five years from his death, and then to be used in the founding of a hospital " for the care of sick persons in indigent circumstances residing in the county of Suffolk, Mass." The members of the Corporation were Alexander Corcoran, Edward D. Codman, Eben S. Draper, Henry S. Howe, Walter Hunnewell, Lawrence H. H. Johnson, and William R. Trask. Augustus Hemenway was elected a member in 1909, when the charter was amended, and the Governor of the Commonwealth appointed John P. Reynolds and Erving McD. Garfield as State members of the Corporation for three and six years respectively.

### Admirable Procedure.

The Corporation had to face prolonged litigation in the Courts before its funds were free to be expended for the purpose named in Mr. Brigham's will. The late Dr. J. S. Billings, of New York, was first engaged to give expert advice and to work out, in connection with the site conditionally purchased, a ground plan for the buildings, which, when completed, would constitute the new hospital. A guaranty fund was raised, and Dr. Billings was sent to Europe. After an extended tour, in which he visited the chief hospitals there, he framed the ground plan that was finally used. Six architects were invited to compete. and their plans were submitted, without names or means of identification, to a committee of experts: Dr. J. S. Billings, Professor F. W. Chandler, and Dr. H. B. Howard, formerly of the Massachusetts General Hospital, who was appointed superintendent on April 2, 1908, when he went to Europe and spent the summer in the study of hospital construction and management. In the result the plans submitted by Messrs. Codman and Despradelle were accepted. The contracts for the construction of the hospital buildings were signed on August 1, 1911, and they were practically completed by July 1913. The first patient was admitted early in 1913, and the highest number of patients under treatment at any one time has been 173 patients.

The levels of the site constituted no small difficulty at the beginning, and the way these have been successfully overcome reflects credit upon the late Dr. J. S. Billings, the present superintendent, Dr. Howard, and the architects. The chief feature of the plan of the ward blocks is its pavilion. The two devoted to surgery have the operation theatres apart from the ward pavilions and so placed as to facilitate the arrangement of moving the patients from the room to the ward and *vice versa*. The main corridor runs from the rotunda in the administration building through the whole length of the site. Proceeding from the rotunda of the ward section we pass the block for private patients on the left and the domestic building on the right. The former contains a large number of comfortable rooms, with the necessary offices for the reception of paying patients. It does not, however, call for special comment. The domestic building is exceedingly well planned, and has on the ground floor accommodation for the staff of all grades, exclusive of the nurses; each section of the staff has separate dining accommodation. The whole of the food served is delivered in the large service room in the centre of the domestic building, and is thence distributed to the various dining-rooms set aside for the accommodation of the medical and each other section of the working staff.

There are seven ward pavilions, of which four have been erected—namely, C, D, E, and F; A, the pavilion for paying patients, is also completed and in occupation, whilst B, intended to be a future ward, and G, a special ward, have yet to be erected. The superintendent's house occupies a position behind G on the right of the corridor, but wholly distinct from it. On leaving the ward section and entering the grounds at the end of the main corridor we pass the superintendent's house on the right, and also the laundry, situated in a building which includes a small department for the manufacture of soap for the supply of the whole hospital, and the sleeping accommodation for the male domestic staff. Immediately adjacent on the left of the laundry and distinct from it is the shop, an erection of considerable size, where ample machinery, stores, stock, and equipment have been provided for the mechanical department. It has proved, Dr. Howard reports, very satisfactory because the means are there to solve any ordinary mechanical problem in machinery, electricity, carpentry, or painting. Many special instruments have been constructed according to the specifications of members of the staff.

### The Hospital Spirit.

We published a full account, with plans of the wards and hospital buildings proper, in our issue of November 4, 1916, p. 101, and may content ourselves with expressing here the satisfaction and pleasure it gave us to spend a whole day in this hospital with Dr. Howard, and to realise to the full the thorough and excellent way in which he has converted his many ideas, the result of a great number of fruitful years as superintendent of various hospitals, into practice at the Peter Bent Brigham Hospital. The difficulties of the site have been tackled with courage and success. No attempt has been made at architectural effect, but the hospital as it stands is the product of much practical experience, and as a centre of up-to-date modern hospital work in all its branches, it does credit to those responsible for the plan. A further and notable feature is the evident aim of the present administration to emphasise as a real measure of the utility of the hospital the importance of the work that is done for the patients. Whatever may be said, it is clear to us that no one can with justice declare that the patients, their requirements, comfort, hygienic provision, treatment, housing, isolation when necessary, protection and care, have been anywhere or in any sense overlooked throughout the hospital. The evidence abundantly present justifies Dr. Howard's claim that the hospital's utility is splendidly demonstrated and convincingly proved by the work that has been done for its patients. This notable feature is brought home to the visitor who studies the rotunda in the administration building and notices the abundant and suitable accommodation there provided for everyone who has business in the hospital, from the visitors of the superintendent to those of the humblest patient within its walls. This rotunda is an old feature in hospital construction, and its great advantage is that it enables an eye to be kept upon the whole working of the business of the hospital at all times, whilst it facilitates the prompt giving of information and the readiest possible communication between members of the staff and the wards, between the patients and their friends, and the general conduct of the hospital business throughout all its departments.

The ward pavilions are original in design and novel in their arrangement. On the third floor there is an outdoor ward of the best type, on the second floor there is a fine terrace upon which the patients' beds can be wheeled out from the wards when the weather is suitable and the doctors sanction this course. The porch, with eight beds, has ample floor space, and the arrangement whereby the concrete on which the beds stand can be warmed to a suitable temperature when desired is novel. On the second floor the two small wards, one for one and one for two beds, are popular with the patients and satisfactory, whilst the larger wards on the main floor, which contain six and fourteen beds, with two small wards of two and one bed respectively, and the adjacent accommodation, including the class-room, clinical laboratory with duty-room, two bathrooms, a linen-room, assistants' accommodation, and the necessary offices, are attractive from the patients' and teachers' point of view. The only doubt we have is to whether the minor accommodation in regard to lavatory, etc., will be found adequate unless the large majority of the patients are habitually confined to bed. The terrace on the main floor of pavilion C, like that on the third floor, and the pavilion accommodation throughout the ward sections everywhere, constitutes a special feature of first importance and value.

### Full-time Work.

This is one of the earliest of American hospitals to introduce what has been known as full-time work, which means continuous service in the hospital by members of the staff instead of having changes several times a year. The position of surgeon-in-chief was conferred on Dr. Harvey Cushing and that of physician-in-chief on Dr. Henry A. Christian. Both commenced their duties in the summer of 1912 under a temporary arrangement with Harvard University, these gentlemen occupying respectively the Chairs of Surgery and Medicine in the Harvard Medical School.

A large outdoor department has been established, and by arrangement with the Boston Dispensary many of its cases are transferred to the wards of the hospital. A social service department has been established, and has proved most beneficial in supplementing the usual hospital work by following the patients on their discharge to their own homes.

### Statistical Reports.

A notable feature of the first annual report is the attention paid to the tables of statistics; these are only provisional, but in the second report, which is now due, they are to be perfected and will be looked forward to with genuine interest. In some hospitals tables of statistics are entirely omitted, a course unworthy of a reputable hospital. The need of the moment is an entirely satisfactory medical and surgical table of statistics. Dr. Howard rightly insists that a report without such statistics may, rightly or wrongly, lay the hospital open to the charge of concealing unpleasant facts, whilst a report in which only the important parts of the work are considered can give no one an idea of the full scope of the hospital work. To argue that such reports have had little interest in the past only shows that they were not properly drawn up. Dr. Howard's medical colleagues in their reports rightly emphasise that there is no uniformity in the various reports of hospitals. Those issued by the Peter Bent Brigham Hospital are offered, not as models or solutions of these problems, but as the best that the staff could do at the present time. Dr. Howard hopes that in the near future it will be possible to have the clinical reports of all the Boston hospitals following one scheme, and that eventually all hospitals may adopt one general plan.

## Figures and Tables

**Figure f1:**